# Transplantation of Isl1^+^ cardiac progenitor cells in small intestinal submucosa improves infarcted heart function

**DOI:** 10.1186/s13287-017-0675-2

**Published:** 2017-10-16

**Authors:** Lingjun Wang, Elizabeth M. Meier, Shuo Tian, Ienglam Lei, Liu Liu, Shaoxiang Xian, Mai T. Lam, Zhong Wang

**Affiliations:** 1 The First Affiliated Hospital, Guangzhou University of Chinese Medicine, Guangzhou, 510405 China; 2 Department of Cardiac Surgery, Cardiovascular Center, The University of Michigan, Ann Arbor, MI 48109 USA; 3 Department of Biomedical Engineering, Wayne State University, Detroit, MI 48201 USA; 4 Faculty of Health Sciences, University of Macau, Macau SAR, China; 5 Cardiovascular Research Institute, Wayne State University, Detroit, MI 48201 USA

**Keywords:** Cardiac progenitor cell, Small intestinal submucosa, Heart regeneration, Myocardial infarction

## Abstract

**Background:**

Application of cardiac stem cells combined with biomaterial scaffold is a promising therapeutic strategy for heart repair after myocardial infarction. However, the optimal cell types and biomaterials remain elusive.

**Methods:**

In this study, we seeded Isl1^+^ embryonic cardiac progenitor cells (CPCs) into decellularized porcine small intestinal submucosa extracellular matrix (SIS-ECM) to assess the therapeutic potential of Isl1^+^ CPCs and the biocompatibility of SIS-ECM with these cells.

**Results:**

We observed that SIS-ECM supported the viability and attachment of Isl1^+^ CPCs. Importantly, Isl1^+^ CPCs differentiated into cardiomyocytes and endothelial cells 7 days after seeding into SIS-ECM. In addition, SIS-ECM with CPC-derived cardiomyocytes showed spontaneous contraction and responded to β-adrenergic stimulation. Next, patches of SIS-ECM seeded with CPCs for 7 days were transplanted onto the outer surface of infarcted myocardium in mice. Four weeks after transplantation, the patches were tightly attached to the surface of the host myocardium and remained viable. Transplantation of patches improved cardiac function, decreased the left ventricular myocardial scarring area, and reduced fibrosis and heart failure.

**Conclusions:**

Transplantation of Isl1^+^ CPCs seeded in SIS-ECM represents an effective approach for cell-based heart therapy.

**Electronic supplementary material:**

The online version of this article (doi:10.1186/s13287-017-0675-2) contains supplementary material, which is available to authorized users.

## Background

Myocardial infarction (MI) is among the leading causes of death globally. In the United States, the overall prevalence of MI is 3.0% among adults older than age 20 years, and MI caused over 1.1 million deaths in 2014 [1]. Although many patients survive acute MI, most of them subsequently develop heart failure, which likely results from left ventricular (LV) remodeling. Previous studies have confirmed that ischemic cardiomyocyte death, myocardial fibrosis, and inflammation are characteristic pathological features of cardiac remodeling after MI [2, 3]. Cardiomyocyte loss after MI has long been considered irreversible, with the heart lacking sufficient regenerative capacity [4–6].

Heart transplantation is the only curative treatment for heart failure, but is subject to significant limitations, such as lack of available donors and problems with immune rejection or infection [7]. Stem cell-based therapies have been proposed as a potential strategy to promote regeneration of the injured myocardium. Previous studies had shown that cells were lost rapidly after direct injection into the myocardium, intracoronary injection, or intravenous injection [8–13]. A promising alternative strategy has been to use biomaterials to provide an adequate three-dimensional scaffold for transplanted cells, thereby increasing cell viability and even producing better cardiac tissue models in vivo [14, 15]. Various types of cells have been combined with different materials for cardiac repair and generation of new myocardium, including induced pluripotent stem cells, cardiomyocytes (CMs), and cardiac progenitor cells (CPCs) [16–18]. Bone marrow stem cells promote angiogenesis and modulate inflammation in a paracrine fashion, but cannot directly achieve the regeneration of functional myocardium [19–21]. Many studies have shown that CMs are the ideal cell type for production of cardiac tissue, but it is very difficult to isolate human CMs for clinical application. Therefore, CMs derived from pluripotent stem cells could be the most feasible cell source for generation of cardiac tissue in combination with biomaterials, but purified CMs cannot form vascularized tissue or grafts when seeded into biomaterials.

In contrast, embryonic CPCs can be differentiated from embryonic stem/induced pluripotent cells and then manipulated to undergo differentiation into various types of cardiac cells, including CMs, vascular endothelial cells, and smooth muscle cells. Therefore, CPCs could be the ideal cell source for engineering of vascularized tissues or grafts [22, 23]. Embryonic CPCs can be identified by a specific marker (Isl1) and have been isolated from the second heart field [24, 25]. As bona-fide cardiac progenitors, Isl1^+^ CPCs may be a more suitable cell source for cardiac tissue engineering and heart regeneration [26–32].

Decellularized porcine small intestinal submucosa extracellular matrix (SIS-ECM) is a xenogeneic biodegradable scaffold with properties that allows its use as a biomaterial [33, 34]. Several studies have demonstrated that SIS-ECM promotes tissue repair in regenerative applications such as wound healing [33, 35, 36]. SIS-ECM can serve as a scaffold that provides adequate three-dimensional support for transplanted cells, potentially sustaining cell survival and promoting proliferation [33, 37]. In addition, it does not trigger an antigenic response and can induce regeneration of native tissue after transplantation in vivo [38]. Therefore, in this study we aim to determine the therapeutic potential of Isl1^+^ CPCs seeded in SIS-ECM to repair MI heart.

## Methods

### Culture, differentiation, and characterization of cells

Mouse embryonic stem cells (ES) were cultured according to the protocol reported previously and differentiation into CPCs was performed according to a previously published protocol with some modifications [39]. In brief, mES cells were digested with TrypLE (Invitrogen) and suspended in differentiation medium 1 (DM1: IMDM supplemented with Ham’s F12, bovine serum albumin, B27, N2 (Invitrogen), monothioglycerol, and vitamin C (Sigma-Aldrich)) at a density of 75,000 cells/ml for culture in a Petri dish for 48 hours. The embryoid bodies (EBs) that formed were then harvested, dissociated, and cultured in DM1 supplemented with 5 ng/ml VEGF, 5 ng/ml Activin A, and 0.8 ng/ml BMP4 (R&D Systems, Minneapolis, MN, USA) at a density of 100,000 cells/ml in a Petri dish for 40 hours. The EBs were collected again, dissociated, and cultured in 0.1% gelatin-coated cell culture dishes in differentiation medium 2 (DM2: StemPro-34 SFM supplemented with vitamin C, glutamine (Invitrogen), monothioglycerol (Sigma-Aldrich), 5 ng/ml VEGF, 10 ng/ml bFGF, and 25 ng/ml FGF10 (R&D Systems)) for 32 hours to generate Isl1^+^ CPCs. The Isl1^+^ CPCs were collected and seeded in SIS-ECM or culture plate, and then cultured in DM2 medium without growth factors (no VEGF, bFGF, or FGF10) for 7-day differentiation. The cells were characterized by immunofluorescence staining using the standard protocol. Fluorescence images were obtained with an Olympus BX53 fluorescence microscope (Olympus Corporation, Tokyo, Japan).

### Assessment of the biocompatibility of SIS-ECM with Isl1^+^ CPCs

Porcine SIS-ECM was kindly donated by CorMatrix Cardiovascular, Inc. (Atlanta, GA, USA). Isl1^+^ CPCs were prestained with Dil (Invitrogen) and were seeded directly into 24-well plates or into SIS-ECM before culture in DM2. The cell density in SIS-ECM was 1.0 × 10^5^/mm^3^. Fluorescence images were captured at 24 hours after seeding, and then cck8 reagent (Dojindo Molecular Technologies, Rockville, MD, USA) was added to the wells and culture was continued for another 2 hours. An aliquot (200 μl) of culture medium was transferred to a 96-well plate and the absorbance was measured at 450 nm using a microplate reader. After 24 hours of culture, some of the SIS-ECM/cell constructs were fixed overnight at 4 °C with 2.5% phosphate-buffered glutaraldehyde (Sigma-Aldrich), and post-fixed for 1 hour with 1% osmium tetroxide (Sigma-Aldrich). The samples were then rinsed three times with PBS, dehydrated through a graded ethanol series, and dried using hexamethyldisilazane (Sigma-Aldrich). Processed SIS-ECM/cell constructs were cut open at the center, sputter-coated with gold, and imaged at 10 kV using a Philips XL30 FEG scanning electron microscope (Philips, the Netherlands) to detect cell adhesion. On days 1 and 7 of culture, SIS-ECM/cell constructs were fixed with 4% paraformaldehyde for 10 min, and then were placed in 5% sucrose-PBS for 1 hour and in 15% sucrose-PBS for another 1 hour. Specimens were embedded in CRYO-OCT Compound (Andwin Scientific, Tryon, NC, USA), frozen at –20 °C, and cut into 10-μm sections. Cells in the SIS-ECM/cell constructs were stained by DAPI (1:10,000) at 24 hours after seeding. On day 7, the differentiated cells in culture plates and frozen sections of SIS-ECM/cell constructs were fixed with 4% paraformaldehyde for immunofluorescence staining by the standard protocol.

### Animal models

This study was approved by the Committee for Animal Research of the University of Michigan and was performed in accordance with the recommendations of the American Association for the Accreditation of Laboratory Animal Care.

### Induction of MI and transplantation of SIS-ECM patches seeded with Isl1^+^ CPCs

MI was induced in nude (NU-Foxn1nu) mice (Charles River Laboratories, Wilmington, MA, USA) by permanent ligation of the left anterior descending coronary artery (LAD). Then 3.6 mm^3^ of SIS-ECM (depth 3 mm, width 3 mm, height 0.4 mm) was seeded with 3.6 × 10^5^ Isl1^+^ CPCs and incubated in DM2 medium without growth factors for 7 days before transplantation. Fibrin gel (EVICEL® Fibrin Sealant (Human); Ethicon, Somerville, MA, USA) was applied to the edges of SIS-ECM patches containing Isl1^+^ CPC-differentiated cardiac cells with DM2 medium for attachment of the patches to the infarcted region of the left ventricle after induction of MI or to the left ventricle of normal hearts.

### Histological analyses

Histological studies were performed by standard protocols. Briefly, mice were sacrificed and their hearts were perfused with 20% KCl, fixed with zinc fixative solution (BD Pharmingen), and dehydrated with 30% sucrose. After embedding in paraffin, the samples were sectioned and processed for Masson’s trichrome staining. Images were captured with an Aperio (Leica Biosystems, Buffalo Grove, IL, USA) and infarct size was measured using Image J software.

### Real-time polymerase chain reaction analysis

Total RNA was extracted from cells in culture plates, cells in SIS-ECM, or the left ventricles of mice using an RNAeasy Mini kit (Qiagen, Valencia, CA, USA), and 1 μg of total RNA was converted to cDNA with reverse transcriptase in a 10-μl reaction mixture. We tested the products of each RT reaction by regular PCR to confirm reverse transcription. Real-time PCR was carried out with a SYBR Green PCR Master Mix kit (Invitrogen, Carlsbad, CA, USA) and the 7300 Sequence Detection system (ABI).

### Echocardiography

Echocardiography was performed at 28 days after MI using a Vevo 770 system (Visualsonic, Toronto, Canada). Using M-mode tracings, the following parameters were measured by an investigator blinded to the treatment of the mice: the LV ejection fraction (EF), cardiac output, end-diastolic volume, and LV end-diastolic and end-systolic dimensions (LVEDD and LVESD, respectively). In addition, LV fractional shortening (LVFS) was calculated according to the following equation:

$$ \mathrm{LVFS}=\left[\left(\mathrm{LVEDD}\hbox{--} \mathrm{LVESD}\right)/\mathrm{LVEDD}\right]\times 100. $$

### Statistical analysis

Results are presented as the mean ± SD. Data were analyzed by one-way analysis of variance followed by Fisher’s least significant difference test. Differences were considered significant at *p* < 0.05.

## Results

### Isl1^+^ CPCs were easily attached to and proliferated normally in SIS-ECM

**Fig. 1:**
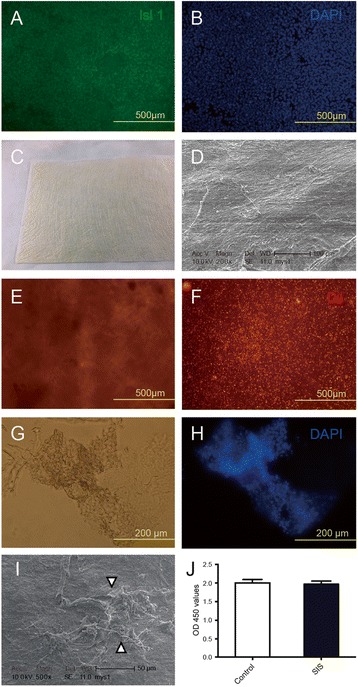
Isl1^+^ CPCs remain viable and attach to the extracellular matrix of the patch material (SIS-ECM). **a**, **b** Isl1^+^ CPCs. **c** Extracellular matrix (SIS-ECM) patch material. **d** Scanning electron micrograph of SIS-ECM. **e**, **f** Viable Isl1^+^ CPCs prestained by Dil in SIS-ECM obtained by bright-field (**e**) and fluorescence (**f**) microscopy. **g**, **h** Images of frozen sections of Isl1^+^ CPCs seeded into SIS-ECM obtained by bright-field (**g**) and fluorescence microscopy (**h**). **i** Scanning electron microscopy shows attachment of Isl1^+^ CPCs (arrowhead) in SIS-ECM. **j** CPC proliferation rate in SIS-ECM compared to standard culture (control) measured using the Cell Counting Kit-8 (CCK-8) assay. SIS small intestinal submucosa

Isl1^+^ CPCs were obtained as described previously. Differentiation of mES cells was induced by a published EB-based cardiac differentiation protocol with minor modifications. Isl1^+^ CPCs were identified by immunofluorescence staining (Fig. 1a, b). Proliferation Isl1^+^ CPCs (Additional file 1: Figure S1) were harvested and seeded into the SIS-ECM patches (Fig. 1c, d) at 1.0 × 10^5^ cells/mm^3^. In order to investigate whether the patches supported cell survival, viable Isl1^+^ CPCs prestained with Dil (Invitrogen) were observed by bright-field and fluorescence microscopy (Fig. 1e, f) at 24 hours after seeding. DAPI staining of frozen sections also showed that Isl1^+^ CPCs remained viable in SIS-ECM patches (Fig. 1g, h). To investigate attachment of Isl1^+^ CPCs to the SIS-ECM patch material, patches were seeded with Isl1^+^ CPCs and scanning electron microscopy was performed, revealing that CPCs spread over and became attached to the patches (Fig. 1i). In addition, the proliferation rate of CPCs in SIS-ECM and in culture plates (control) was compared by the Cell Counting Kit-8 (CCK-8) assay, revealing no significant difference of proliferation between SIS-ECM and control culture (Fig. 1j). In contrast, as a control, we found that it was more challenging to seed ESC-derived CMs into SIS-ECM patches, which could be partially due to the relatively larger size and irregularity of the cell shape of the CMs.

### Isl1^+^ CPCs differentiated into CMs and endothelial cells in SIS-ECM

**Fig. 2:**
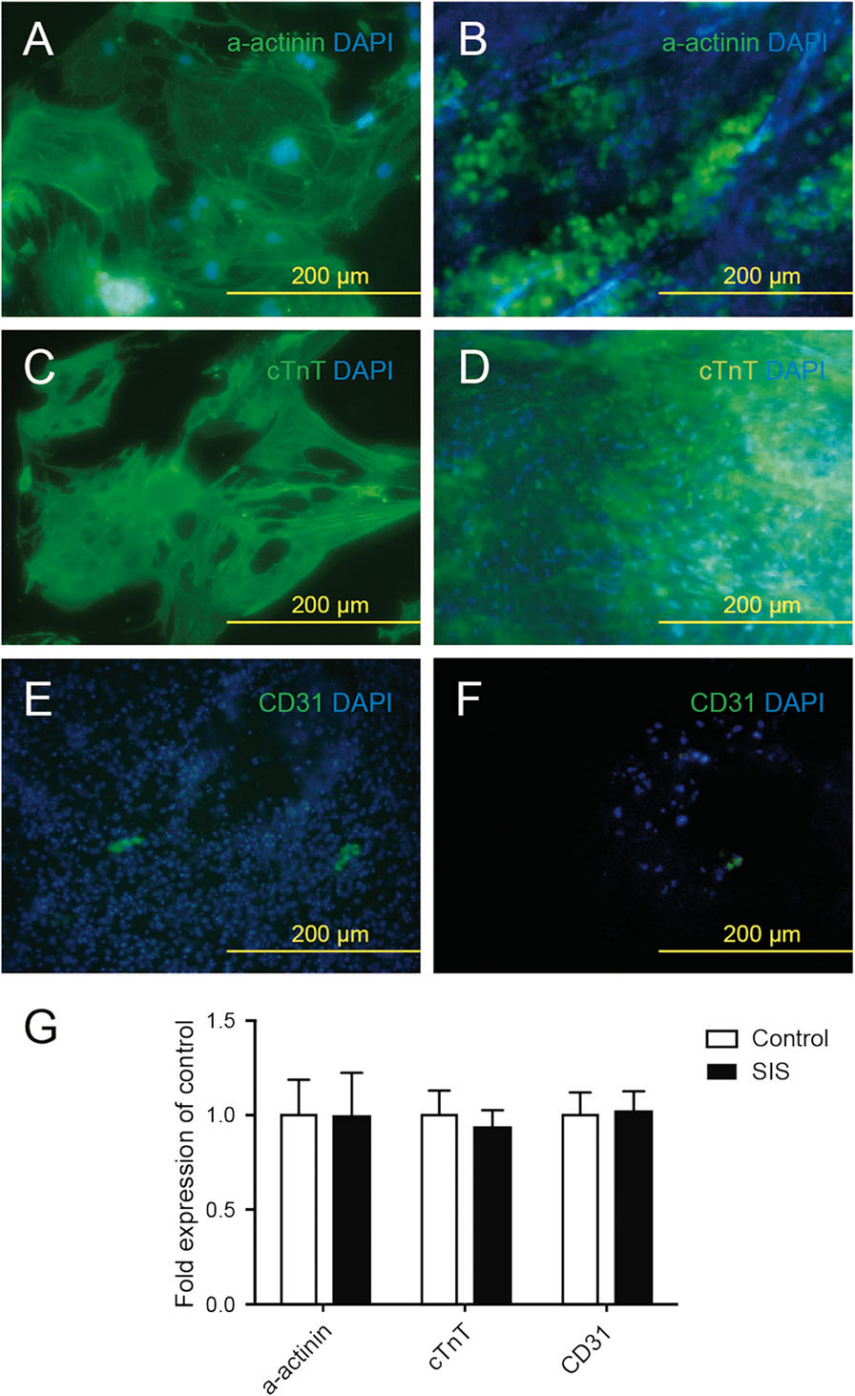
Isl1^+^ CPCs differentiate into CMs and endothelial cells after seeding into SIS-ECM. **a**, **c** Isl1^+^ CPC-derived CMs (α-actinin-positive and cTnT-positive) in culture obtained by fluorescence microscopy. **b**, **d** Isl1^+^ CPC-derived CMs (α-actinin-positive and cTnT-positive) in SIS-ECM obtained by fluorescence microscopy. **e** Isl1^+^ CPC-derived endothelial cells (CD31^+^) in culture obtained by fluorescence microscopy. **f** Isl1^+^ CPC-derived endothelial cells (CD31^+^) in SIS-ECM obtained by fluorescence microscopy. **g** Relative quantitative RNA expression of α-actinin, cTnT, and CD31 by CPC-derived CMs or endothelial cells growing in SIS-ECM compared to standard culture plates. SIS small intestinal submucosa

To evaluate the multilineage differentiation potential of Isl1^+^ CPCs, we investigated the transition of cells seeded into the SIS-ECM from CPCs to CMs and endothelial cells. Immunofluorescence revealed differentiation of Isl1^+^ CPCs into CMs (α-actinin-positive and cTnT-positive cells; Fig. 2b, d) and endothelial cells (CD31-positive cells; Fig. 2e) at 7 days after seeding into SIS-ECM or culture plates (Fig. 2a, c, e). Real-time PCR showed that there were no significant differences of α-actinin, cTnT, and CD31 mRNA expression in cells between SIS-ECM and control culture.

### Isl1^+^ CPCs seeded in SIS-ECM formed a multilayered cardiac tissue-like structure and responded to β-adrenergic stimulation

**Fig. 3:**
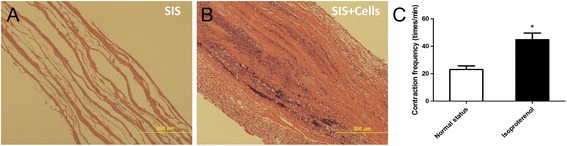
Structure of SIS-ECM seeded with CPCs for 1 week and response to β-adrenergic stimulation. **a**, **b** Cross-sectional images (HE staining) of SIS-ECM (**a**) and SIS-ECM seeded with CPCs after 1 week (**b**). **c** Contraction rate of SIS-ECM seeded with CPCs before and after the addition of 10 μM isoproterenol on day 8 of culture. Data expressed as mean ± SD. *n* = 5. *p < 0.05 vs basal state. SIS small intestinal submucosa

HE-stained cross-sections of SIS-ECM showed a multilayered structure (Fig. 3a), and indicated that a multilayered cardiac tissue-like cell sheet (Fig. 3b) was generated by seeding Isl1^+^ CPCs into SIS-ECM. To investigate the response of the cardiac tissue-like cell sheet to β-adrenergic stimulation, 10 μM isoproterenol was added to the culture medium, resulting in a reversible significant increase in the frequency of contraction (Fig. 3c).

### Transplantation of SIS-ECM patches seeded with CPCs reduced myocardial scarring after MI

**Fig. 4:**
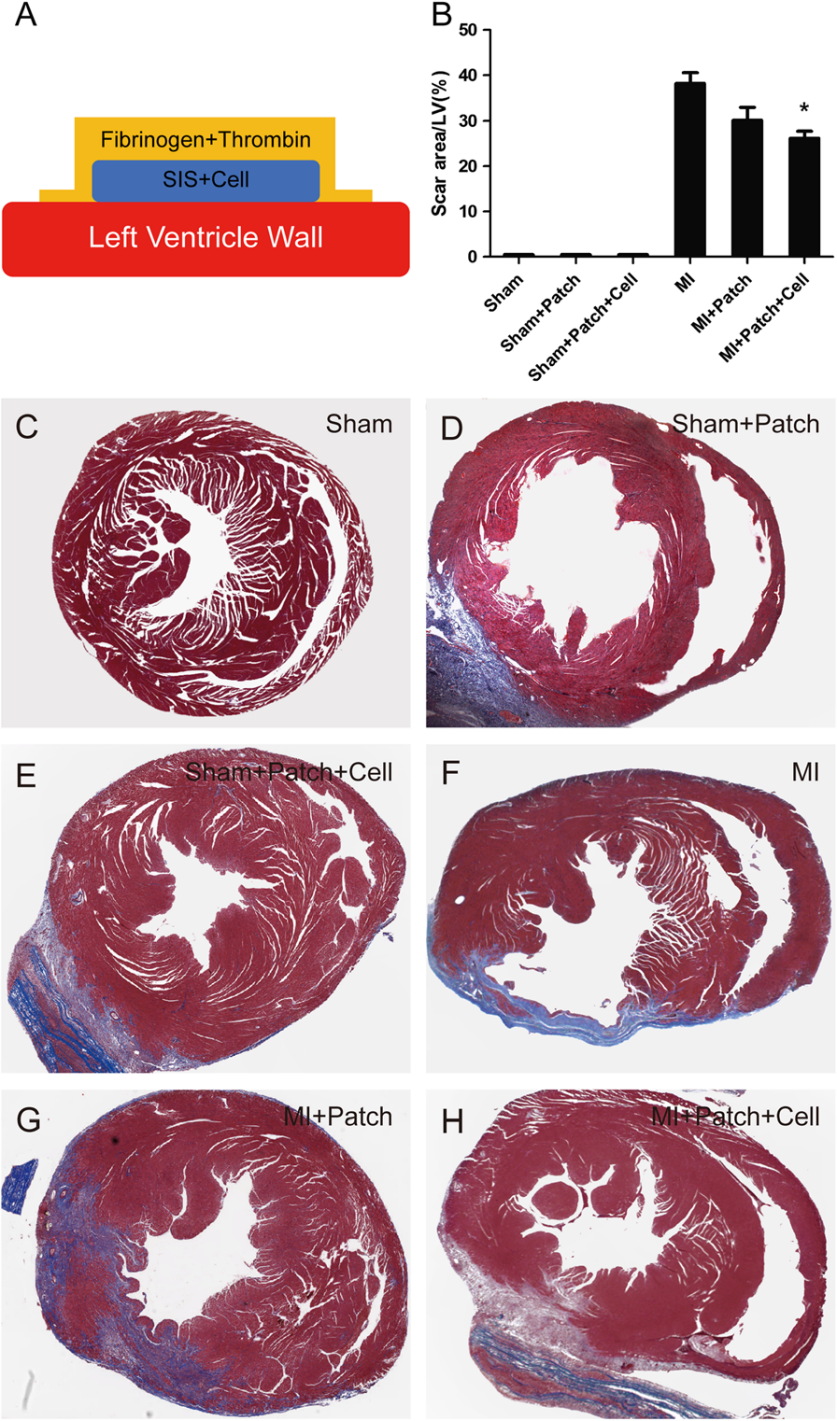
Implantation of SIS-ECM seeded with CPCs reduced myocardial scarring. Schematic view showing attachment of SIS-ECM-CPC patch to the heart with fibrin gel (**a**). Percent area of myocardial scarring in mice with MI. Data are mean ± SD obtained from Masson’s trichrome slides (**b**). Data expressed as mean ± SD. *n* = 6. *p = 0.003 vs MI mice. Representative images of mouse hearts (Masson’s trichrome staining) for Sham group (**c**), Sham + Patch group (**d**), Sham + Patch + Cell group (**e**), MI group (**f**), MI + Patch group (**g**), and MI + Patch + Cell group (**h**). LV left ventricular, MI myocardial infarction, SIS small intestinal submucosa

The formation of beating multilayered tissue-like structures in SIS-ECM after seeding with CPCs prompted us to examine the patch’s function in heart repair in vivo. SIS-ECM was seeded with Isl1^+^ CPCs and cultured in vitro for 7 days before transplantation. Nude mice (Charles River Laboratories, Wilmington, MA, USA) were used to generate MI by permanent ligation of the left anterior descending coronary artery (LAD); immediately after MI, SIS-ECM and SIS-ECM-CPC patches were transplanted to the surface area of infarcted region of the left ventricle. Gel was applied to the edges of SIS-ECM patches to improve attachment of the patches to the hearts. Mice were euthanized on day 28 after transplantation and the hearts were harvested for histological analysis. As shown in Fig. 4, transplantation of SIS-ECM or SIS-ECM-CPC to healthy mice did not affect the heart morphology (Fig. 4, b, compare c to d and e). Transplantation of SIS-ECM to MI heart appeared to have modest effect on reducing the scar area (Fig. 4, b, compare g to f; *p* = 0.061). In contrast, SIS-ECM with CPC seeding significantly reduced the scar area compared with the MI group (Fig. 4, b, compare h to f; *p* = 0.003). This indicated that transplantation of SIS-ECM patches seeded with CPCs maximally reduced infarct size after MI.

### Transplantation of SIS-ECM patches with CPCs improved cardiac function after MI

**Fig. 5:**
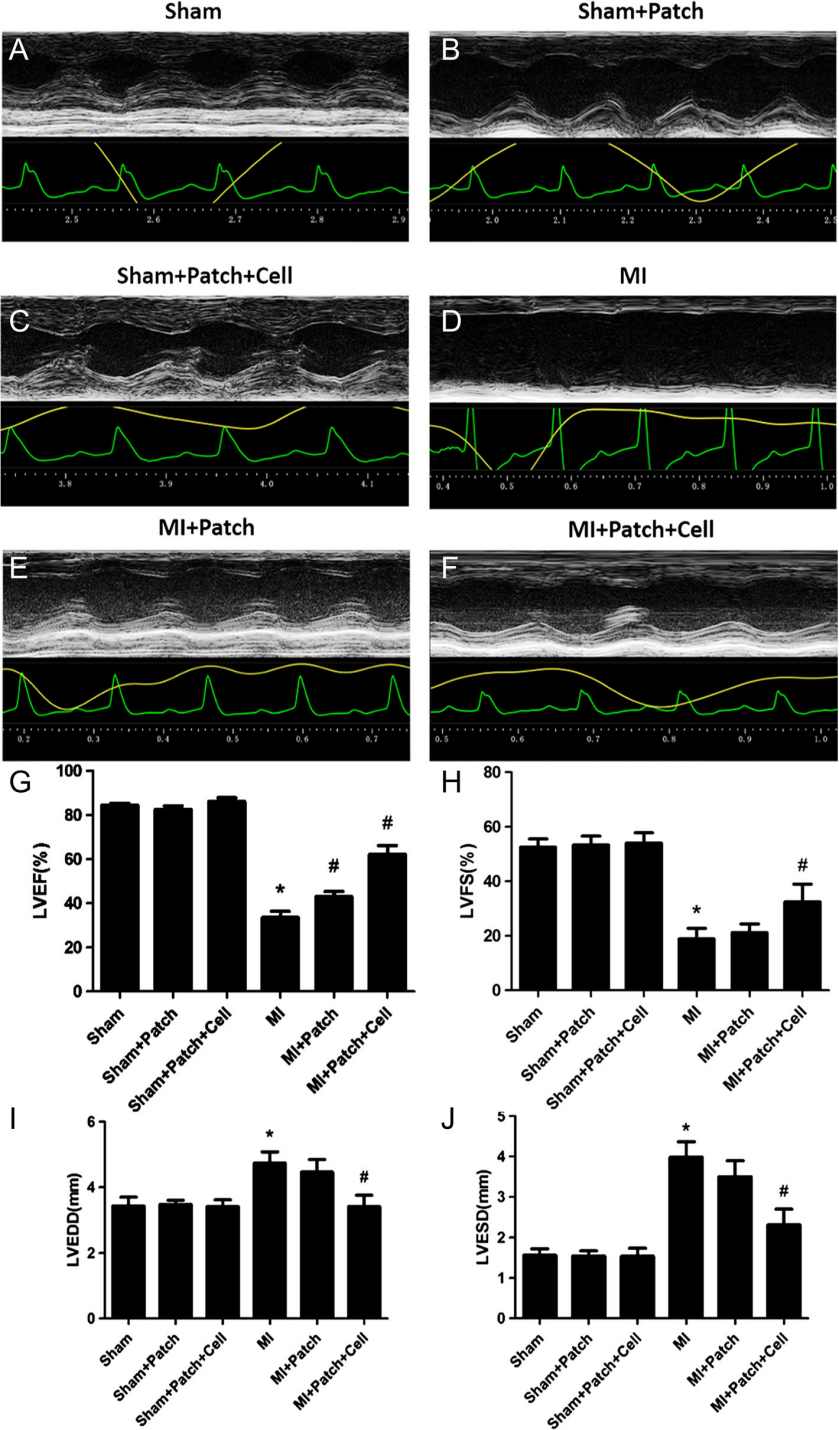
Implantation of SIS-ECM-CPC improved cardiac function in mice with MI. Representative M-mode echocardiographic images obtained in Sham (**a**), Sham + Patch (**b**), Sham + Patch + Cell (**c**), MI control (**d**), MI + Patch (**e**), and MI + Patch + Cell (**f**) groups. Quantitative cardiac function analysis of LVEF (**g**), LVFS (**h**), LVEDD (**i**), and LVESD (**j**) 4 weeks after permanent LAD occlusion of the six groups. Data expressed as mean ± SD. *n* = 6. MI + Patch + Cell vs sham mice, *p < 0.001; MI + Patch + Cell vs MI mice, *#p* < 0.004. Modest or no statistical significance was detected between MI + Patch and MI groups. *n* = 6. MI + Patch vs MI mice, LVEF, #*p* = 0.034. LVEF left ventricular ejection fraction, LVFS left ventricular fractional shortening, MI myocardial infarction

Echocardiographic examination was performed 28 days after transplantation of SIS-ECM-CPCs. There were no significant differences among the Sham + Patch, Sham + Patch + Cell, and Sham groups (Fig. 5, a, c, g, h), suggesting that cell-seeded patch implantation did not affect the function of normal hearts. In mice with MI, echocardiography showed a significant decrease of LVEF (33.48 ± 6.36%) and LVFS (18.88 ± 3.81%) and a significant increase of LVEDD (4.74 ± 0.34%) and LVESD (3.99 ± 0.38%) compared with Sham mice (Fig. 5, a, d, g–j). Transplantation of the patch alone increased the LVEF modestly in MI mice (Fig. 5, d, e, g). In contrast, transplantation of  Patch + Cell significantly increased the LVEF (62.16 ± 8.96%) and LVFS (32.44 ± 6.54%) and decreased the LVEDD (3.41 ± 0.35%) and LVESD (2.32% ± 0.38%) compared with the MI only mice (Fig. 5, d, f, g–j). These findings suggested that implantation of SIS-ECM seeded with CPC-derived CMs could be an effective strategy to ameliorate LV dysfunction with heart failure after MI.

### Transplantation of SIS-ECM-CPC patches reduced BNP, ANP, Collagen I, Collagen III, and TGF-β mRNA expression in the LV myocardium after MI

**Fig. 6:**
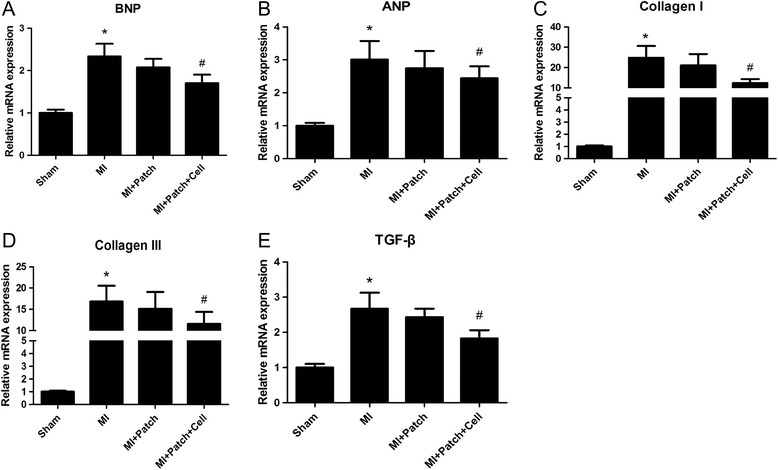
Implantation of SIS-ECM-CPC reduced myocardial BNP, ANP, Collagen I, Collagen III, and TGF-β mRNA expression in mice with MI. **a** BNP, **b** ANP, **c** Collagen I, **d** Collagen III, and **e** TGF-β mRNAs were detected by real-time PCR using cDNAs obtained from the whole LV myocardium of sham mice, MI mice, MI + Patch mice, and MI + Patch + Cell mice, and expression was normalized for that of GAPDH. Data expressed as mean ± SD. *n* = 6. *p < 0.05 vs sham mice, *#p* < 0.05 vs MI mice. MI myocardial infarction

Real-time quantitative PCR analysis indicated that expression of BNP, ANP, Collagen I, Collagen III, and TGF-β mRNA was increased in the LV myocardium of mice with MI-induced heart failure compared with sham mice mice (Fig. 6a–e). Consistent with our echocardiography findings, implantation of SIS-ECM seeded with CPC-derived CMs reduced BNP, ANP, Collagen I, Collagen III, and TGF-β mRNA expression in the LV myocardium (Fig. 6a–e). These data demonstrate that the transplantation of SIS-ECM-CPC significantly prevented heart failure, improved cardiac function, and reduced myocardial fibrosis in mice after MI.

## Discussion

In this study, we investigated whether transplantation of SIS-ECM seeded with Isl1^+^ CPCs could improve cardiac function and alleviate myocardial damage after MI. To generate a high yield of Isl1^+^ CPCs from mES cells, we employed modified EB-based differentiation based on previous protocols [39]. In order to determine whether Isl1^+^ CPCs could survive in SIS-ECM and attach to the biomaterial, we first investigated the viability and attachment of Isl1^+^ CPCs 24 hours after seeding into SIS-ECM. The findings suggested that SIS-ECM provide a suitable environment for growth and act as a scaffold for Isl1^+^ CPCs. We compared differentiation of Isl1^+^ CPCs into CMs and endothelial cells in SIS-ECM with differentiation under normal culture conditions. Our data indicated no significant differences in the differentiation of CMs and endothelial cells between SIS-ECM and standard culture. This suggested that Isl1^+^ CPCs could effectively differentiate into CMs and endothelial cells after seeding into SIS-ECM, allowing utilization for engineering of vascularized cardiac tissue and cardiac tissue regeneration (Figs. 1, 2, and 3).

In the present study, CPCs were seeded into porcine SIS-ECM, and 7 days after seeding we could observe the alignment of CPC-derived CMs along the longitudinal axis of the SIS fibers. In addition, we observed the spontaneous contraction of SIS-ECM seeded with CPC-derived CMs. The contraction rate of the SIS-ECM/CPC-derived CM constructs was significantly increased by short-term β-adrenergic stimulation with isoproterenol. These findings suggested the formation of a functional patch with contractile activity of CMs.

We believe that our studies represent significant progress compared to a previous study in which a myocardial patch was generated [40]. In the previous study, neonatal rat CMs were seeded to decellularized porcine SIS to form CM sheets adjacent to the SIS layers [40]. The SIS-ECM-CM patch can beat in a somewhat nonsynchronous contraction pattern and preserve heart function after transplantation. However, it is very difficult to isolate human CMs for clinical applications and the seeding of CMs appears very challenging. In particular, it is very inefficient for CM cells to be seeded inside the SIS-ECM. In contrast, embryonic Isl1^+^ CPCs are much easier to be seeded into SIS-ECM. In our studies, we generated a functional spontaneous contracting patch by seeding Isl1^+^ CPCs into porcine SIS-ECM. Our studies suggest an attractive alternative cell source (human ISL1^+^ CPCs) in future clinical studies.

The major finding of the present study was that transplantation of SIS-ECM-CPCs showed considerable therapeutic potential. To avoid secondary damage, SIS-ECM-CPC patches were attached to the outer surface of the infarcted region of the heart using fibrin gel instead of sutures (Fig. 4a). The cells at the surface of the SIS-ECM-CPC patches were in direct contact with the outer surface of the infarcted myocardium after implantation. Four weeks post implantation, the patches were found to be tightly attached to the surface of the host myocardium and remained viable (Fig. 4 and Additional file 2: Figure S2). Moreover, transplantation of SIS-ECM-CPCs decreased the area of myocardial scarring in mice with MI. The results of real-time PCR showed that implantation of SIS-ECM seeded with CPC-derived CMs decreased the expression levels of fibrosis related genes such as TGF-β, Collagen I, and Collagen III. At 4 weeks after permanent ligation, the occurrence of heart failure was confirmed by echocardiography. Transplantation of SIS-ECM-CPCs improved LVEF and LVFS and decreased LVEDD and LVESD at 4 weeks post implantation. In addition, we demonstrated that the transplantation of SIS-ECM-CPC patches did not affect the function of normal hearts (Fig. 5). These data suggested that the patch transplantation method used in this study was safe.

To further determine the therapeutic effect of SIS-ECM-CPC transplantation, we examined the expression of heart failure markers associated with LV systolic dysfunction (Fig. 6). The results of real-time PCR showed that LV myocardial expression of BNP and ANP mRNA was significantly elevated in mice with MI compared with sham mice at 4 weeks after permanent ligation of the LAD. In addition, LV myocardial expression of BNP mRNA was significantly lower in mice from the MI + Patch group than in untreated mice with MI. These data suggested that the transplantation of SIS-ECM-CPCs was able to inhibit heart failure and improve cardiac function induced by MI. However, the effect of transplantation of SIS-ECM-CPC patches on arrhythmia after MI was not investigated in this study, and will need to be assessed in large animals in the future.

The therapeutic effect of the SIS-ECM-CPC patch is likely due to a combination of several mechanisms. A patch can provide regional mechanical support after transplantation [41, 42]. In addition, spontaneous beating of the patch could be an advantage, which could increase contractility like a small LV assist device [43]. In contrast, patches lacking spontaneous contraction could be a burden to the host heart. Furthermore, stem cell transplantation can attenuate inflammatory response, inhibit fibrosis and stimulate cell proliferation through a paracrine effect [44–47]. The observed decrease of gene expression involved in fibrosis and heart failure (Fig. 6) is consistent with this notion. Future studies will reveal the details of these possible mechanisms, including how the beating rate and rhythm of SIS-ECM-CPC can be coordinated with the host beating rhythm initiated by the sinoatrial node.

## Conclusion

Our studies demonstrated that transplantation of small intestinal submucosa seeded with Isl1^+^ cardiac progenitor cells significantly alleviated myocardial damage and improved cardiac function following myocardial infarction in mice.

## Additional files

Additional file 1: Figure S1. Showing the proliferation rate of Isl1^+^ cells by staining with mitotic marker ki67. (TIF 862 kb)

Additional file 2: Figure S2. Showing detection of Isl1^+^ CPC-derived cell viability in SIS-ECM patches 28 days after transplantation. Frozen sections of mouse hearts subjected to myocardial infarction and transplantation of SIS-ECM-CPC patches before (**A–C**) and after fixation by 4% paraformaldehyde (**D**) obtained by bright-field (**A**) and fluorescence microscopy (**B–D**). (TIF 6084 kb)

## Abbreviations

CM: Cardiomyocyte
CPC: Cardiac progenitor cell
EB: Embryoid body
ESC: Embryonic stem cell
MI: Myocardial infarction
SIS-ECM: Small intestinal submucosa extracellular matrix
