# Supplementary figures and images for: Transplantation of Isl1+ cardiac progenitor cells in small intestinal submucosa improves infarcted heart function
Source: Stem Cell Res Ther. 2017 Oct 16;8:230. doi: 10.1186/s13287-017-0675-2 (PMC5644064; doi:10.1186/s13287-017-0675-2)

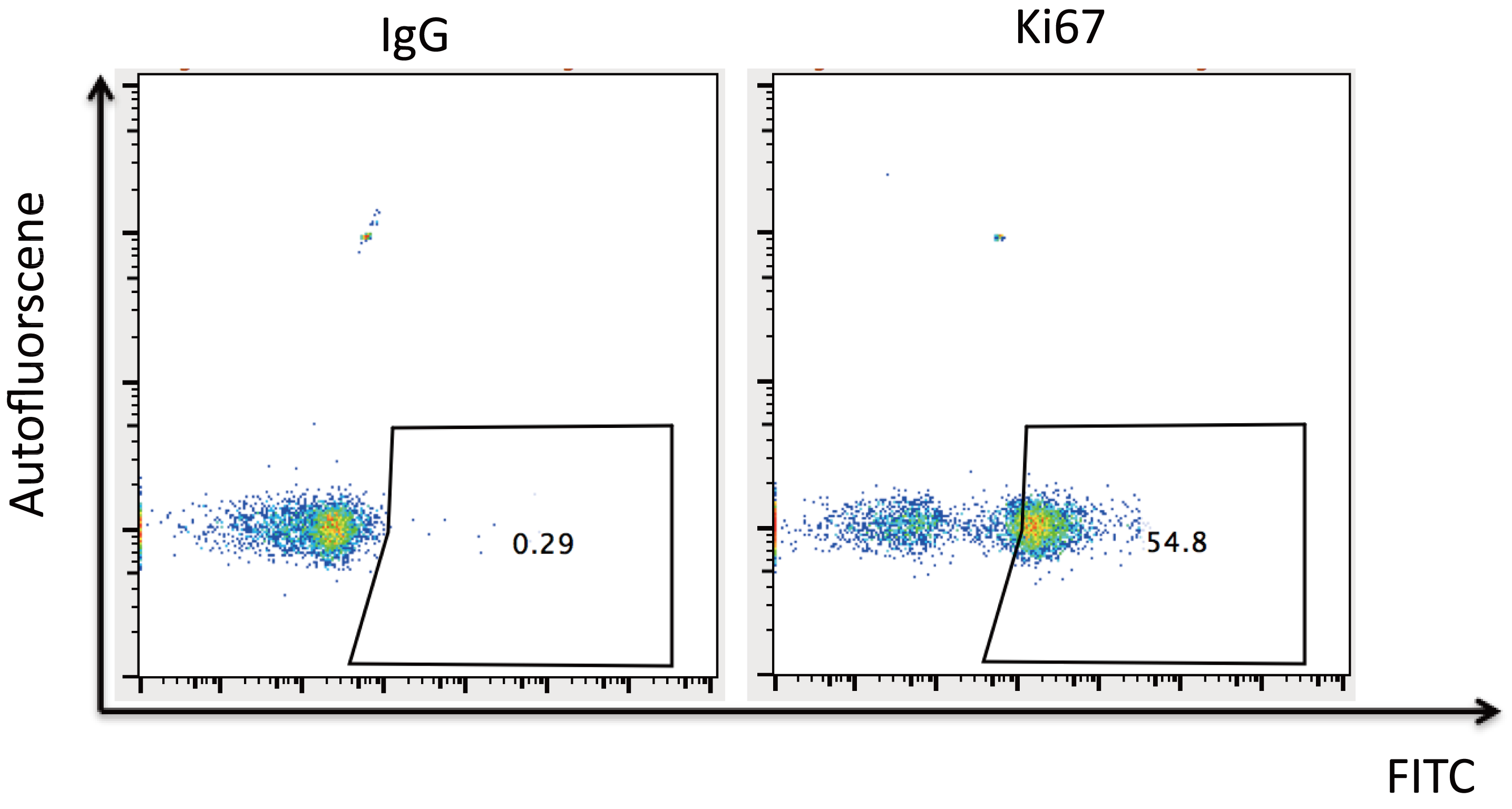

Supplement: Supplementary file 1 — Showing the proliferation rate of Isl1+ cells by staining with mitotic marker ki67. (TIF 862 kb) [file 13287_2017_675_MOESM1_ESM.tif]

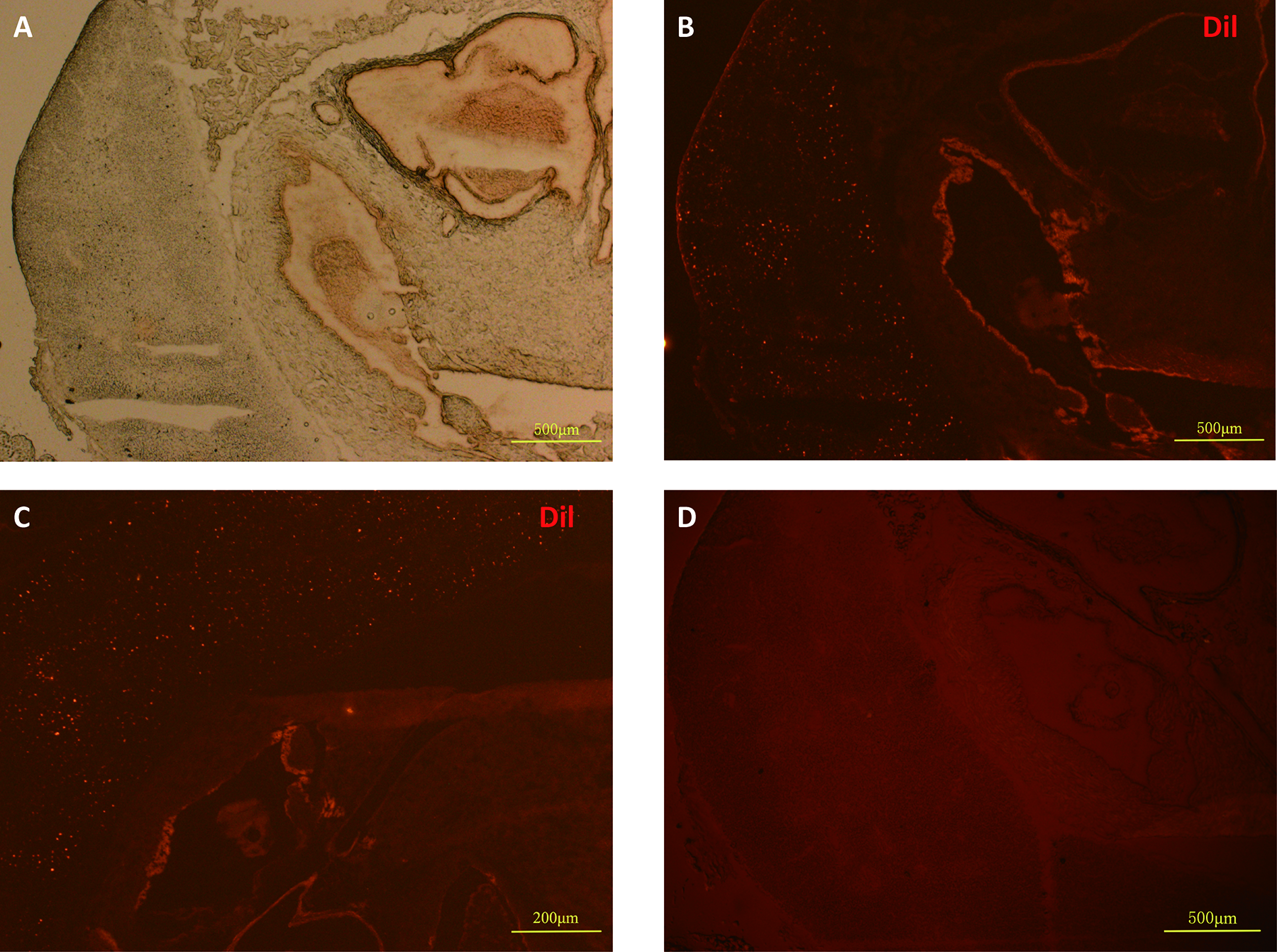

Supplement: Supplementary file 2 — Showing detection of Isl1+ CPC-derived cell viability in SIS-ECM patches 28 days after transplantation. Frozen sections of mouse hearts subjected to myocardial infarction and transplantation of SIS-ECM-CPC patches before (A–C) and after fixation by 4% paraformaldehyde (D) obtained by bright-field (A) and fluorescence microscopy (B–D). (TIF 6084 kb) [file 13287_2017_675_MOESM2_ESM.tif]
